# Supplementary figures and images for: Morphological Neuron Classification Using Machine Learning
Source: Front Neuroanat. 2016 Nov 1;10:102. doi: 10.3389/fnana.2016.00102 (PMC5088188; doi:10.3389/fnana.2016.00102)

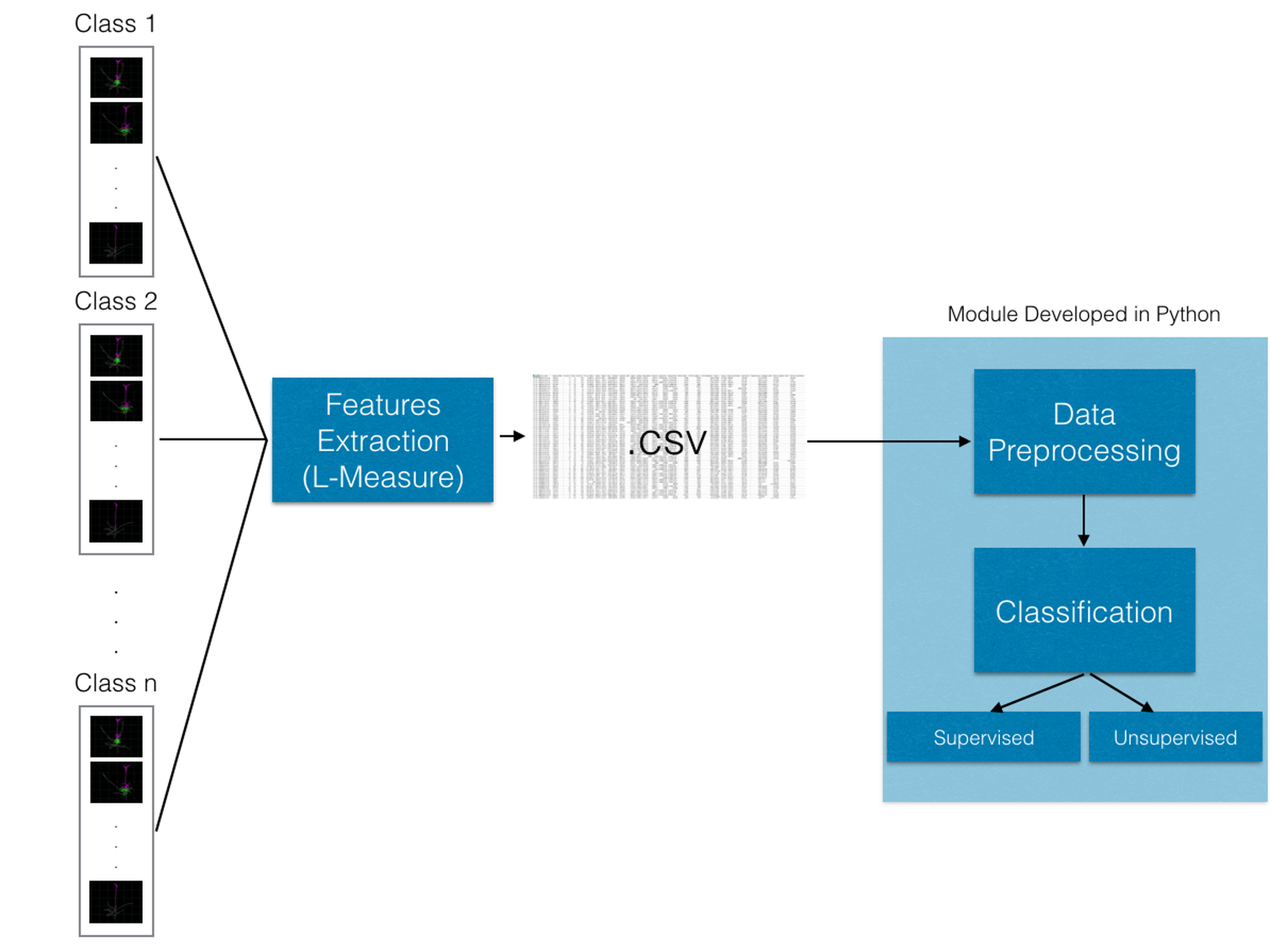

Supplement: FIGURE S1 — Block Diagram of the python pipeline. [file Image_1.JPEG]
